# HazChemNet: A Deep Learning Model for Hazardous Chemical Prediction

**DOI:** 10.3390/ijms26199288

**Published:** 2025-09-23

**Authors:** Nan Zhang, Hexiang Qiu, Hongxia Cai, Zhiru Li, Yutong Li, Zinan Li, Lijuan Qi, Hongju Du, Yan Pan, Haiming Jing, Junyu Ning, Bo Xian, Shan Gao

**Affiliations:** 1 Beijing Key Laboratory of Diagnostic and Traceability Technologies for Food Poisoning, Beijing Center for Disease Prevention and Control, Beijing 100013, China; tyzhangnan@hotmail.com (N.Z.); zinan_li@sina.com (Z.L.); jgxpig@126.com (L.Q.); skydu2008@163.com (H.D.); tabtab1122345@163.com (H.J.); njy_med@hotmail.com (J.N.); 2 Laboratory of Aging Research, School of Medicine, University of Electronic Science and Technology of China, Chengdu 610056, China; qiuhexiang@163.com (H.Q.); ccaijiu@163.com (H.C.); lizhiru_neverland@163.com (Z.L.); yutongli06@163.com (Y.L.); yanpan@zohomail.com (Y.P.); 3 School of Public Health, Capital Medical University, Beijing 100069, China

**Keywords:** deep learning, molecular fingerprints, decision support systems, public safety

## Abstract

The identification of hazardous chemicals is critical for mitigating environmental and health risks, yet existing methods often lack efficiency and accuracy. This study presents HazChemNet, a deep learning model integrating attention-based autoencoders and mixture-of-experts architectures, designed to predict chemical hazardousness from molecular structures. The study utilized a dataset of 2428 hazardous compounds from China’s 2015 hazardous chemical list. Features were derived from molecular fingerprints and physicochemical descriptors, with external validation on 52 unseen chemicals achieving 92.3% accuracy for hazardous and 84.6% for non-hazardous classifications. Experimental validation using *C. elegans* assays confirmed model predictions for critical compounds. Ablation studies confirmed hydrogen bonding features as pivotal predictors, alongside molecular fingerprints. This work bridges the gap between AI-driven innovation and chemical safety, offering a transformative tool for sustainable industrial practices and proactive risk management in a rapidly evolving global landscape.

## 1. Introduction

With the rapid acceleration of global industrialization, hazardous chemicals have found increasingly widespread applications in various sectors such as manufacturing, agriculture, pharmaceuticals, and energy [1,2,3]. These chemicals, due to their high efficiency and diverse functionalities, have significantly contributed to economic development and technological advancements. However, the improper management of hazardous chemicals during production, storage, transportation, and use can lead to severe environmental pollution, ecological damage, and threats to human health [3,4,5]. Frequent incidents such as chemical leaks, explosions, and fires not only result in substantial economic losses but also pose significant challenges to social stability and public safety [6,7]. Therefore, the scientific and accurate prediction and assessment of the properties and potential risks of hazardous chemicals have become crucial for ensuring safe production in the chemical industry and protecting the environment [8,9,10].

In this context, the classification, management, and risk assessment of hazardous chemicals are of paramount importance. The Catalogue of Hazardous Chemicals (2015 Edition) in China serves as a national standard document that systematically lists and categorizes various hazardous chemicals, providing extensive information on physical and chemical properties, as well as hazard indicators [11]. This data forms a foundation for related research and management activities. However, with the increasing variety and complexity of chemicals, traditional manual analysis and assessment methods are no longer sufficient to meet the demands for efficiency and accuracy [12,13]. Consequently, leveraging modern information technology, particularly deep learning and other artificial intelligence (AI) methods, for the prediction and classification of hazardous chemicals has become a focal point of current research [14,15,16].

In recent years, deep learning techniques have achieved remarkable success in areas such as image recognition, natural language processing, and drug discovery [17,18,19]. Their powerful data processing and pattern recognition capabilities offer new possibilities for building predictive models for hazardous chemicals [20,21,22,23]. China’s Catalogue of Hazardous Chemicals (2015 Edition) serves as a critical regulatory foundation for national chemical safety management, governing the production, transportation, and storage of over 3800 hazardous substances to mitigate industrial and environmental risks [24].

Despite its pivotal role, the molecular structure information of these chemicals—particularly standardized SMILES notations—has never been systematically compiled, leaving a significant gap in machine-readable chemical databases [25,26,27,28]. By organizing and integrating molecular structure information (including comprehensive SMILES representations) from single-component compounds listed in the catalogue, this study provides the first complete computational dataset derived from China’s authoritative hazardous chemical registry.

Given the complexity of chemical hazard prediction, traditional machine learning approaches often fall short in handling the non-linear and multifaceted relationships between molecular features and chemical hazards. In this study, we introduce the Attention Mechanism and Mixture of Experts (MoE) model as core components of our predictive framework, designed to address these challenges. The Attention Mechanism allows the model to focus on the most relevant parts of the chemical structure, capturing the subtle dependencies between descriptors that traditional methods might miss. Additionally, MoE is a specialized model that divides the problem into multiple experts, each handling a specific subset of the data, which allows the system to focus on distinct chemical patterns.

This study aims to develop a deep learning-based classification model for predicting hazardous chemicals using the molecular structure information from the Catalogue of Hazardous Chemicals (2015 Edition). Through data organization and integration combined with deep learning models, we seek to achieve efficient and precise predictions of hazardous chemical properties. The establishment of this model will provide scientific decision support for chemical companies, optimize the management processes of hazardous chemicals, and offer technical support for policy-making and emergency response by governmental bodies [29,30]. Furthermore, the research findings have broad application prospects, extending to fields such as environmental monitoring, public safety, and healthcare, contributing to the achievement of sustainable development goals [31,32].

**Figure 1:**
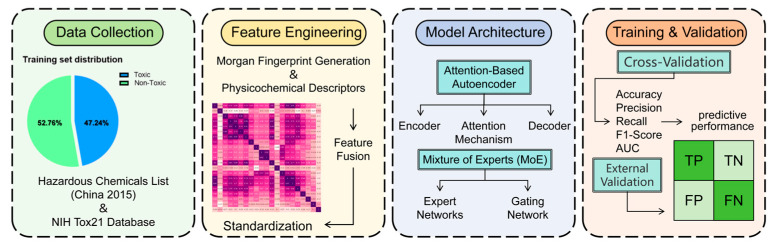
Overview of the HazChemNet framework. The workflow encompasses data collection, feature engineering, hybrid model architecture, and training/validation procedures.

To operationalize these objectives, we developed HazChemNet, a deep learning model combining attention-based autoencoders and a MoE (Figure 1). The following sections detail the dataset construction, feature engineering, and model architecture designed to achieve precise hazardousness prediction. Furthermore, the model’s predictions were experimentally validated through targeted in vivo toxicity assays using *Caenorhabditis elegans* (*C. elegans*), providing empirical confirmation of its hazard classifications.

## 2. Results

### 2.1. Distribution Analysis of Molecular Descriptors

**Figure 2:**
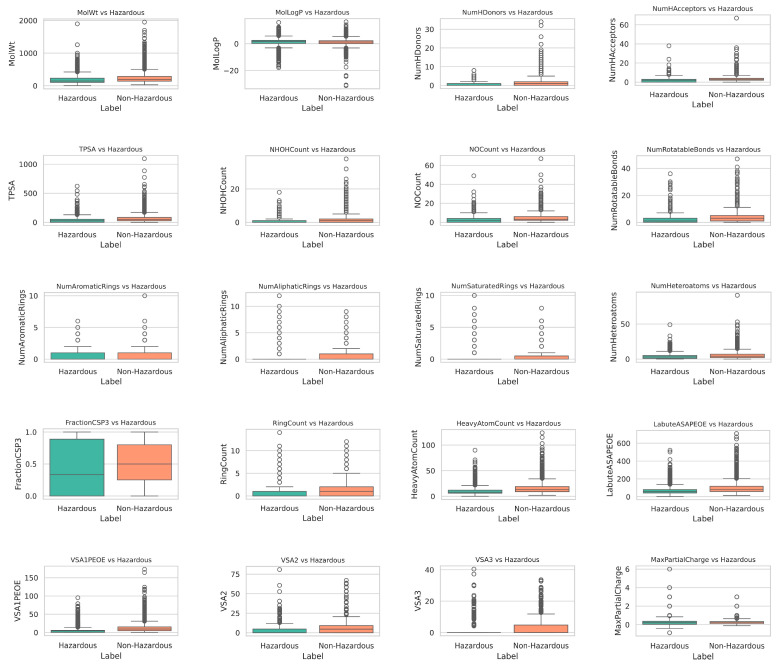
The box plot of 20 molecular descriptors for hazardous and non-hazardous chemicals.

To validate the discriminative power of the selected molecular descriptors, we analyzed the distribution of 20 key descriptors between hazardous and non-hazardous chemicals. As shown in Figure 2, the box plots reveal that the differences between hazardous and non-hazardous chemicals are not particularly large, indicating that they occupy similar regions within the chemical space.

However, some distributional differences can still be observed. For instance, descriptors like MolLogP (lipophilicity) and MolWt (molecular weight) show slight but noticeable disparities. Hazardous chemicals tend to exhibit higher median MolLogP values, suggesting stronger hydrophobic interactions, which may correlate with a greater potential for bioaccumulation. Similarly, MolWt distributions indicate that hazardous chemicals could be slightly larger, which might influence their interaction with biological systems.

We constructed a correlation matrix to explore the relationships between the hazardous and non-hazardous chemical categories and the 20 selected molecular descriptors. As illustrated in Figure S1, no strong correlations were observed between the two groups and the individual descriptors. This lack of significant associations can be attributed to the inherent complexity of the hazardous category, which likely encompasses a diverse range of molecular characteristics. These unique and heterogeneous features do not correlate strongly with any single molecular descriptor, making it difficult to identify a simple, one-to-one relationship.

The absence of prominent correlations underscores the challenge of predicting chemical hazard status based solely on individual descriptors. Given this, the hybrid expert model employed in our predictive framework proves to be highly appropriate. Given these complexities, traditional models may struggle to handle the multifaceted nature of chemical hazard prediction. This is where the Hybrid Expert Model (MoE) and Attention Mechanism come into play.

**Figure 3:**
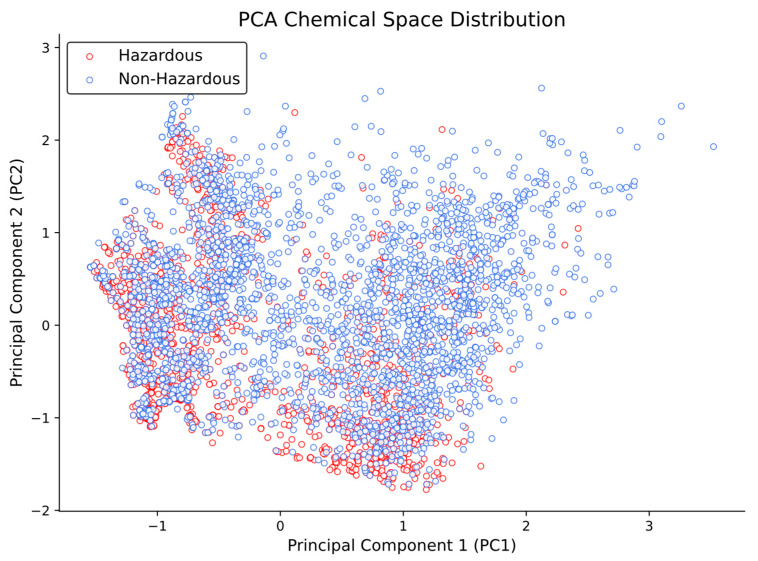
PCA of the 20 selected molecular descriptors for hazardous and non-hazardous chemicals. The plot illustrates that both groups largely overlap within the same chemical space, with no clear clustering or separation between hazardous and non-hazardous categories.

Despite these subtle differences, the Principal Component Analysis (PCA) results (Figure 3) show that both hazardous and non-hazardous chemicals are located in the same, or very similar, chemical space, without a clear separation between the two groups.

### 2.2. Model Performance

The model’s performance was evaluated using 5-fold cross-validation, with each fold independently trained five times to assess stability against inherent stochasticity (e.g., weight initialization and data shuffling). Table 1 provides a detailed comparison of the proposed HazChemNet model against traditional machine learning algorithms, including Support Vector Machine (SVM), Random Forest, and Logistic Regression, across multiple performance metrics with an accuracy of 91.9 ± 1.3%. In contrast, SVM, Random Forest, and Logistic Regression exhibit lower accuracies.

As shown in Table S1, all folds exhibited highly consistent results, with minimal standard deviations (SD) across repeated runs. The mean accuracy ranged from 90.8% (Fold 4) to 93.0% (Fold 3), while precision and recall varied narrowly between 87.9–90.2% and 92.8–95.0%, respectively. The overall metrics further validated robustness, achieving a mean accuracy of 91.9 ± 1.3%, F1-score of 91.4 ± 1.3%, and AUC of 92.9 ± 1.1%. Critically, the low standard deviations (≤2.0% for all metrics) within individual folds—such as Fold 1 (Accuracy: 92.3 ± 1.2%) and Fold 3 (Recall: 95.0 ± 1.0%), underscore the model’s stability across training iterations. These results demonstrate reliable generalizability and minimal sensitivity to random initialization, positioning the model as a robust tool for hazardous chemical prediction.

### 2.3. External Validation of the Model’s Effectiveness and Reliability

To evaluate the effectiveness and reliability of the model, we conducted an external validation using a set of chemicals that were not included in the training dataset. Specifically, we selected 26 hazardous chemicals from sources outside the List of Hazardous Chemicals (2015 Edition) and 26 non-hazardous chemicals provided by the National Institutes of Health (NIH).

For the 26 hazardous chemicals, the model correctly predicted 24 as harmful and incorrectly classified 2 as non-harmful. This results in an accuracy of 92.3% and a false negative rate of 7.7% (or 2.7% if considering the proportion of the total 26 chemicals).

For the 26 non-hazardous chemicals, the model correctly predicted 22 as non-harmful and incorrectly classified 4 as harmful. This results in an accuracy of 84.6%. The detailed results of the external validation are provided in Supplementary Materials S3.

These results demonstrate that the model exhibits high predictive accuracy and robustness when applied to external datasets, further validating its effectiveness and reliability in classifying both hazardous and non-hazardous chemicals. The external validation provides additional confidence in the model’s generalization capabilities and its potential for practical applications in chemical safety assessment.

### 2.4. Ablation Study

To evaluate the contribution of each feature to the model’s predictive capability, we conducted an ablation study by systematically removing individual features and comparing the resulting performance metrics. The results, summarized in Table 2, demonstrate the impact of each feature on model accuracy, precision, recall, and F1 score.

**Figure 4:**
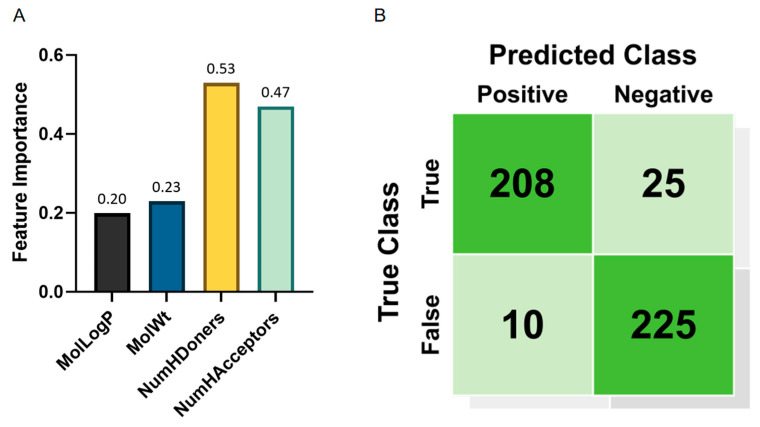
(**A**) The relative importance of physicochemical descriptors in predicting chemical hazardousness. Hydrogen bond donors (NumHDonors) and acceptors (NumHAcceptors) exhibit the highest contributions, followed by molecular weight (MolWt) and lipophilicity (MolLogP). (**B**) Confusion matrix of the model. The *x*-axis represents the predicted labels, and the *y*-axis represents the true labels. The four quadrants represent the correctness of the classification. The top-left quadrant shows the number of correctly predicted “Non-Hazardous” samples (208), the top-right quadrant shows the number of non-Hazardous samples misclassified as “Hazardous” (25), the bottom-left quadrant shows the number of hazardous samples misclassified as “Non-Hazardous” (10), and the bottom-right quadrant shows the number of correctly predicted “Hazardous” samples (225). The confusion matrix provides an overview of the model’s performance in the “Hazardous” and “Non-Hazardous” classification tasks.

As shown in Figure 4A and Table 2, hydrogen bond donors (NumHDonors) exhibit the highest contribution.

The results indicate that the hydrogen bond donors (NumHDonors) and hydrogen bond acceptors (NumHAcceptors) are the most critical features, as their removal leads to the largest performance degradation. For instance, excluding NumHDonors reduces accuracy by 2.1% and F1 score by 2.1%, while removing NumHAcceptors results in a 2.6% drop in accuracy. The Morgan fingerprint also plays a significant role, with its absence causing a 6.9% decline in accuracy, highlighting the importance of molecular topological information. These findings align with the feature importance analysis and further validate the necessity of integrating both physicochemical descriptors and structural fingerprints for robust hazardousness prediction.

### 2.5. Confusion Matrix

From the confusion matrix (Figure 4B), it can be observed that the model correctly identified 208 non-Hazardous samples, while 25 non-Hazardous samples were misclassified as harmful. For the hazardous samples, the model correctly classified 225, but 10 were incorrectly labeled as non-Hazardous. Overall, the model is more accurate in identifying hazardous samples, with a recall rate of 81%, indicating that the majority of Hazardous samples are correctly identified.

### 2.6. Experimental Validation

To empirically verify the predictive reliability of the HazChemNet model, we conducted acute toxicity assays using *Caenorhabditis elegans* (*C. elegans*, N2 strain). We acknowledge that HazChemNet predicts the general “hazardous property” of a chemical, which is a broad concept. In a biological context, this property is often represented by specific toxicological endpoints. Therefore, we selected mortality in *C. elegans* as the biological endpoint to validate the model’s predictions.

A panel of six compounds was selected based on their prediction scores from HazChemNet to serve as positive and negative validation cases. Compounds with high hazardous scores were designated as the predicted “toxic group”, which included ethyl alcohol, atropine, and lead acetate. Conversely, those with low scores—glycerol, gibberellin, and citric acid—comprised the predicted “non-toxic group”.

Synchronized L4-stage worms (*n* = 50 per group) were exposed to compound concentrations for 24 h at 20 °C. All compounds were prepared in K-M buffer. Controls included K-M buffer blanks and 0.2% DMSO solvent controls. Mortality was quantified through tactile response absence, with LD_50_ values derived via Probit analysis.

**Figure 5:**
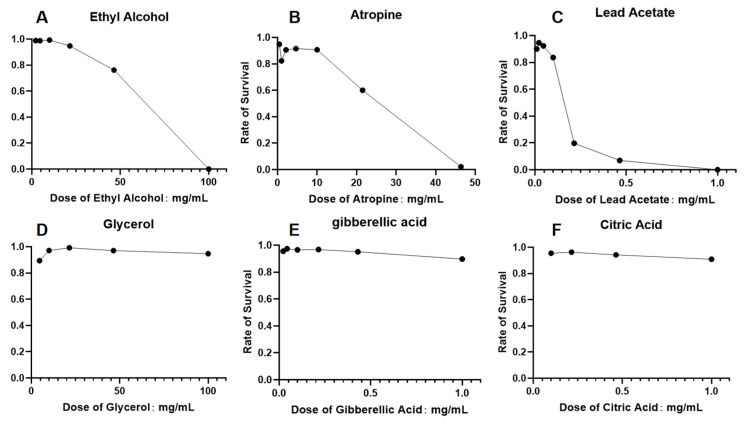
In vivo toxicity validation of model-predicted compounds in *C. elegans*. (**A**) Ethyl Alcohol; (**B**) Atropine; (**C**) Lead Acetate; (**D**) Glycerol; (**E**) Gibberellin; (**F**) Citric Acid.

Results demonstrated complete concordance between model predictions and experimental outcomes. All compounds in the toxic group exhibited dose-dependent lethality (Figure 5A–C) (Table 3).

Conversely, citric acid, gibberellin, and glycerol induced no significant mortality, validating their non-toxic classification (Figure 5D–F).

While this experiment primarily focused on acute lethality as the toxicological endpoint, it serves as a practical and effective means to initially test the predictive validity of HazChemNet. Though acute toxicity is a narrow and short-term measure of chemical hazards, it provides a reproducible and quantifiable biological response. Using *C. elegans* mortality offers a well-established assay for assessing toxicity, allowing us to verify the predictive power of the model within controlled experimental conditions. While the experimental design is not exhaustive and does not capture the full range of potential hazards, it provides a valuable initial validation step and demonstrates that the HazChemNet model can successfully predict the hazardous nature of chemicals based on their molecular descriptors.

## 3. Discussion

### 3.1. Deep Learning for Environmental Safety

The management of hazardous chemicals is a critical challenge in industrial and environmental safety, requiring accurate identification of risks associated with molecular structures [1,2,3]. Despite advancements in computational toxicology, existing studies often lack standardized, machine-readable datasets for region-specific regulatory inventories, particularly in non-Western contexts. China’s Catalogue of Hazardous Chemicals (2015 Edition)—a cornerstone policy document governing over 3800 hazardous substances—has long been hindered by the absence of systematically organized molecular structure data [24].

To address this gap, our study presents the first comprehensive compilation of SMILES notations for all single-component compounds within the catalogue, creating a high-quality, publicly accessible dataset that bridges regulatory frameworks with modern machine learning applications.

Our study presents the design and implementation of a molecular hazardousness prediction architecture that combines an attention-based autoencoder with a MoE model. By introducing an attention mechanism in the autoencoder, the model is able to automatically focus on the most critical features, thereby enhancing the quality of feature representation. The MoE model leverages the collaborative effect of multiple expert networks, dynamically adjusting the weights of each expert based on the input data, which enhances the model’s flexibility and accuracy [20,21]. This integrative approach proved particularly valuable for resolving chemical classification controversies. The experimental results demonstrate that the proposed model achieves excellent predictive performance on the test set, validating the effectiveness of the architectural design. This approach provides a novel perspective for predicting the hazardousness of compounds and holds significant potential for practical applications in chemical safety assessment.

External validation using 26 hazardous and 26 non-hazardous chemicals not included in the training dataset further validated the model’s effectiveness and reliability. The model correctly predicted 24 out of 26 hazardous chemicals as harmful, achieving an accuracy of 92.3% and a false negative rate of 7.7%, with experimental verification extending this rigor to critical compounds including ethanol, atropine, and lead acetate. For the non-hazardous chemicals, the model correctly classified 22 out of 26, resulting in an accuracy of 84.6%. The ablation study confirms that hydrogen bonding capacity (NumHDonors/NumHAcceptors) and molecular topology (Morgan fingerprints) are pivotal for hazardousness prediction. The high recall for hazardous compounds (81%) suggests the model effectively identifies hazardous chemicals, aligning with regulatory priorities.

### 3.2. Strengths of the Model

The model’s capacity to reconcile computational predictions with experimental evidence represents a key advancement. By combining Morgan fingerprints with physicochemical descriptors, it captures both localized structural patterns and global molecular properties, ensuring a rich and balanced feature representation that underpins accurate predictions.

The attention-based autoencoder further refines these features by dynamically prioritizing critical structural elements linked to hazardousness, thereby enhancing interpretability and predictive fidelity. This is synergistically complemented by a MoE framework, which harnesses the collaborative power of specialized neural networks that adaptively adjust their contributions based on input data, significantly improving flexibility and accuracy in handling chemically diverse and structurally complex compounds.

Furthermore, its automation-driven approach streamlines chemical safety assessments, substantially reducing reliance on costly and time-intensive experimental testing, which positions it as a cost-effective and scalable solution for real-world hazardous chemical management [14,15,16].

### 3.3. Limitations of This Study

While the model demonstrates strong predictive performance, there are several areas for improvement and further research.

Firstly, the dataset used in this study has a slight imbalance between positive and negative samples, which can affect the model’s performance. Future work should focus on addressing this imbalance through techniques such as oversampling, undersampling, or the use of weighted loss functions.

Secondly, the current model is relatively complex, which can make it computationally intensive. Future research could explore more efficient architectures or optimization techniques to reduce computational costs without compromising predictive performance. While the current feature set is comprehensive, there may be additional features or representations that could further enhance the model’s performance. Exploring other types of molecular fingerprints, such as ECFP or MACCS, and incorporating more advanced physicochemical descriptors could be beneficial.

While the model shows promise in laboratory settings, its real-world applications need to be explored. Future work should focus on deploying the model in industrial and regulatory settings to assess its practical utility and impact on chemical safety management.

### 3.4. Future Directions

As the volume and variety of chemical data continue to grow, it is essential to ensure that the model can scale and generalize to new and unseen chemicals. This can be achieved by continuously updating the model with new data and fine-tuning it to adapt to different chemical classes and properties.

The model can be integrated into decision support systems for chemical companies, regulatory agencies, and environmental monitoring organizations. This integration will provide real-time predictions and recommendations, enabling more informed and timely decisions.

We can also extend the model to handle multiple tasks, such as predicting different types of hazardousness (e.g., mutagenicity, carcinogenicity, and ecotoxicity), which can provide a more comprehensive risk assessment. Multi-task learning can also help in identifying common patterns and features across different hazardousness endpoints.

As the use of AI in chemical safety assessment becomes more widespread, it is crucial to address ethical and regulatory considerations. Ensuring transparency, fairness, and accountability in the model’s predictions and decision-making processes will be essential for building trust and acceptance among stakeholders.

By automating hazard prediction and reducing reliance on costly experimental testing, this work provides a scalable, cost-effective tool for regulatory compliance and risk assessment. Our integration of China’s authoritative chemical inventory with state-of-the-art deep learning techniques sets a precedent for leveraging region-specific regulatory data to advance global chemical safety efforts. Future studies could extend this framework to multi-component mixtures or integrate real-time monitoring systems, further empowering intelligent hazardous chemical management.

## 4. Materials and Methods

We developed and evaluated a deep learning model for predicting the hazardousness of chemical compounds, addressing the critical need for accurate and efficient safety assessments in various industries. The dataset was constructed by integrating 2428 single-component hazardous chemicals from the 2015 edition of the “List of Hazardous Chemicals” and 2712 non-toxic compounds from the NIH’s Tox21 database, all represented in SMILES format.

To capture both local and global structural information, we utilized Morgan fingerprints and physicochemical descriptors. These features were then combined and standardized to form a comprehensive feature vector. The model architecture combines an autoencoder for dimensionality reduction and a MoE for classification, leveraging the strengths of both methods to enhance predictive performance.

### 4.1. Data Collection and Dataset Construction

We organized and integrated the molecular structures of single-component chemicals from the 2015 edition of the “List of Hazardous Chemicals” to establish a dataset for training a deep learning model for predicting molecular hazardousness. The main steps of this process are as follows:

#### 4.1.1. Data Collection

We collected 2428 single-component hazardous chemicals from the 2015 edition of the “List of Hazardous Chemicals” to form the positive samples in our training set. From the NIH’s Tox21 database, we selected 2712 compounds that did not show any activity in all hazardousness assays as negative samples. The molecular structures of these compounds were represented in SMILES (Simplified Molecular Input Line Entry System) format.

#### 4.1.2. Dataset Splitting

The dataset was split into training, validation, and test sets in a 7:2:1 ratio to ensure that the model could effectively distinguish between harmful and non-harmful compounds.

The training set, consisting of 3598 compounds, is the primary source of data for the model to learn the relationship between the features and labels. The validation set, comprising 1028 compounds, is used for hyperparameter tuning, performance validation, and monitoring the training process.

The test set, with 514 compounds, is used to evaluate the model’s performance on unseen data after the model has been fully trained, serving as an external validation. And the ratio of positive to negative samples in the dataset is 0.9.

#### 4.1.3. Detailed Compound Information

A complete list of the molecular compounds, along with their corresponding SMILES information, is provided in Supplementary Materials S1 (Hazardousness Prediction Model Dataset). The Supplementary Materials include detailed molecular structures and labels for each compound. Positive samples are labeled as 1, and negative samples are labeled as 0.

This structured approach ensures a robust and comprehensive dataset for training and evaluating the deep learning model, enabling accurate predictions of chemical hazardousness.

With the dataset rigorously structured, we proceeded to extract molecular fingerprints and physicochemical descriptors, as detailed below.

### 4.2. Establish Predictive Molecular Fingerprints

Having established the feature extraction and data preprocessing steps, we now delve into the detailed architecture and performance of our model. In this section we will introduce the design of the hybrid model, the training process, and the evaluation of its predictive capabilities.

#### 4.2.1. Calculation of Molecular Fingerprints

In the molecular hazardousness prediction model, we employed Morgan fingerprints (also known as circular fingerprints) to capture the topological structure information of molecules. Morgan fingerprints are a type of graph-based encoding method that effectively captures local structural information within molecules.

The length of the fingerprint was set to 512 bits, meaning each molecule is encoded into a 512-bit vector and the radius of the fingerprint was set to 2, which means that the neighborhood structure within two chemical bonds around each atom in the molecule is considered during the generation of the fingerprint.

#### 4.2.2. Morgan Fingerprint Generation

Morgan fingerprints were generated through an iterative encoding process. First, each atom’s chemical properties (e.g., atom type) were initialized as:

$$E_{i}^{\left(0\right)}=\mathrm{AtomProperties}\left(i\right)$$

where i is the initial feature vector of atom.

Subsequently, the encoding of each atom was updated by aggregating information from its neighboring atoms within a radius of 2 bonds, formalized as:

$$E_{i}^{\left(r\right)}=Hash\left(E_{i}^{\left(r-1\right)}+\sum_{j\in \mathrm{neighbors}\left(i\right)}E_{j}^{\left(r-1\right)}\right)$$

where r is the current radius, $E_{i}^{\left(r-1\right)}$ is the encoding of atom j from the previous step, and Hash is a function that hashes the information to generate the fingerprint.

After this process, each molecule is encoded into a 512-bit fingerprint vector, which represents the topological structure of the molecule.

#### 4.2.3. Extraction of Molecular Descriptors

To more comprehensively characterize the properties of the molecules, we used the RDKit package 2025.3.6. to extract physicochemical descriptors. These descriptors provide global property information about the molecules, further enhancing the model’s understanding of their hazardousness.

The extracted physicochemical properties include: Molecular weight (MolWt), Lipophilicity (MolLogP), Number of hydrogen bond donors (NumHDonors), Number of hydrogen bond acceptors (NumHAcceptors).

These descriptors were chosen to capture global chemical properties that complement local structural patterns from Morgan fingerprints.

#### 4.2.4. Feature Combination

We concatenated the extracted molecular fingerprints and molecular descriptors. The final feature vector has a length of 516, consisting of a 512-bit molecular fingerprint and 4 physicochemical descriptors. This combination retains the local structural features of the molecules while incorporating global physicochemical properties, providing a richer set of features for the model.

Let $f_{\mathrm{Morgan}}\in R^{512}$ be the molecular fingerprint and $d=[MolWt,MolLogP,NumHDoners,NumHAcceptors]\in R^{4}$ be the molecular descriptors. The final feature vector is given by:

$$X=\left[f_{\mathrm{Mra}},d\right]\in R^{516}$$

Standardized features were fed into the model for training. The impact of feature selection and combination on model performance is analyzed in Section 2.1.

#### 4.2.5. Data Standardization

Before inputting the features into the model, all features were standardized. This process scales the features to have a mean of 0 and a standard deviation of 1, which helps to avoid issues caused by differences in the scale of different features and can speed up model convergence and improve training performance. The standardization formula is:

$$x_{i}^{\prime }=\frac{x_{i}-\mu_{i}}{\sigma_{i}}$$

where $x_{i}$ is the original feature value, $\mu_{i}$ is the mean of the feature, and $\sigma_{i}$ is the standard deviation of the feature.

This comprehensive approach ensures that the model is provided with a rich and balanced set of features, enabling accurate and reliable predictions of molecular hazardousness.

These combined features were then fed into a hybrid architecture (Section 4.3) to optimize both dimensionality reduction and classification.

### 4.3. Model Architecture Design

The proposed HazChemNet employs a hybrid deep learning architecture comprising an attention-based autoencoder and a MoE model. This synergistic design optimizes feature representation through dimensionality reduction and attention-guided feature selection, followed by dynamic decision-making via expert networks, thereby enhancing both accuracy and adaptability in hazardousness prediction.

#### 4.3.1. Attention-Based Autoencoder

The autoencoder serves to compress high-dimensional molecular features into a low-dimensional latent space while selectively emphasizing critical features via an attention mechanism. The architecture is structured as follows:

Encoder: The input feature vector x∈R^516^ (concatenated Morgan fingerprints and physicochemical descriptors) is compressed through two fully connected (FC) layers:

$$h_{1}=\sigma \left(W_{1}x\;+\;b_{1}\right),\;z=\sigma (W_{2}h_{1}+b_{2})$$

where $h_{1}$ and z denote hidden and latent representations, respectively, and σ is the ReLU activation function, W_1_ are weight matrices, $b_{1}$, $b_{2}$ are bias vectors.

Attention Mechanism: To prioritize hazardousness-relevant features, normalized attention weights α are computed from the latent code z:

$$u=tanh\left(W_{a}z+b_{a}\right),\alpha_{i}=\frac{exp(u_{i})}{\sum_{j=1}^{z}exp(u_{j})},z^{\prime }=\alpha ⊙z$$

where ⊙ denotes element-wise multiplication. The weighted latent code $z^{\prime }$ accentuates discriminative features for downstream classification, $W_{a}$ is the weight matrix for the attention layer, $b_{a}$ is the bias vector, u is the intermediate attention score vector, i is the normalized attention weight for the i-th feature.

Decoder: The decoder reconstructs $z^{\prime }$ to the original feature space, trained via mean squared error (MSE) loss:

LA=1n∑i=1n(xi−x^i)2

where x^i is the reconstructed feature vector for the i-th sample, and N is the number of samples.

#### 4.3.2. Mixture of Experts (MoE)

The MoE module dynamically integrates predictions from multiple expert networks, each specialized in distinct data patterns, to enhance generalization:

**Expert Networks:** *M* independent FC networks process the attention-refined features $z^{\prime }$, generating intermediate predictions $y_{m}=E_{m}\left(z^{\prime };\theta_{m}\right)$.

**Gating Network:** A gating network assigns weights $g_{m}$ to experts based on $z^{\prime }$:

$$g_{m}=\frac{\exp\left(h_{m}\left(z^{\prime }\right)\right)}{\sum_{m=1}^{M}\exp\left(h_{m}\left(z^{\prime }\right)\right)}$$

where $h_{m}$ denotes the gating network’s output for the m-th expert.

**Final Output:** The model’s prediction is a weighted sum of expert outputs:

$$y=\sum_{m=1}^{M}g_{m}\cdot y_{m}$$

This architecture ensures robust feature representation through the autoencoder and adaptive decision-making via MoE, effectively addressing the complexity and variability of chemical hazardousness data.

### 4.4. Training and Testing of the Hazardous Chemicals Prediction Model

We designed a hybrid model combining autoencoders and a MoE to predict the harmfulness of compounds. We extracted features from the SMILES representation, incorporating 512-bit Morgan fingerprints and molecular descriptors, and used an autoencoder for dimensionality reduction. The low-dimensional features were then fed into the MoE for classification.

By training and testing the model, we achieved strong predictive performance, with precision, recall, and F1 scores for both toxic and non-toxic classes.

These include:

$$\mathrm{Accuracy}=\frac{TP+TN}{TP+TN+FP+FN}$$

where TP is the number of true positives, TN is the number of true negatives, FP is the number of false positives, and FN is the number of false negatives.

$$\mathrm{Precision}=\frac{TP}{TP+FP}$$

Precision measures the proportion of true positive predictions among all positive predictions.

$$\mathrm{Recall}=\frac{TP}{TP+FN}$$

Recall measures the proportion of true positive predictions among all actual positive cases.

$$F1-\mathrm{Score}=2\cdot \frac{\mathrm{Precision}\cdot \mathrm{Recall}}{\mathrm{Precision}+\mathrm{Recall}}$$

The F1-Score is the harmonic mean of precision and recall, providing a balanced measure of both.

$$\mathrm{AUC}-\mathrm{ROC}=\int_{0}^{1}\mathrm{TPR}\left(FPR\right)\;d\left(FPR\right)$$

See Supplementary Materials S2, Model Design Specification.

#### 4.4.1. Model Training and Testing

**Figure 6:**
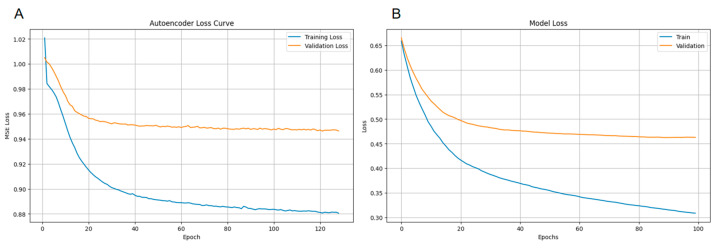
(**A**) Loss curves of the autoencoder on the training and validation sets. The *x*-axis represents the number of training epochs, and the *y*-axis represents the mean squared error (MSE) loss. The blue curve represents the training loss, and the orange curve represents the validation loss. (**B**) Loss curves of the MoE model on the training and validation sets. The *x*-axis represents the number of training epochs, and the *y*-axis represents the loss value. The blue curve represents the training loss, and the orange curve represents the validation loss.

As shown in Figure 6A, the training loss of the autoencoder decreases continuously with increasing iterations and stabilizes after 120 epochs. The validation loss, on the other hand, decreases rapidly in the initial stages and then remains relatively stable, indicating that the model is continuously optimizing on the training set while maintaining a stable performance on the validation set. This suggests that the model does not exhibit significant overfitting.

The training was conducted on a server with dual nVidia Tesla P40 GPUs. In addition to the GPUs, the server was equipped with an Intel Xeon E5-2630 v4 CPU, which offered solid multi-core performance, and 128 GB of RAM, ensuring smooth data processing and efficient model training. The operating system used was Ubuntu 22.04, which facilitated the setup of the deep learning framework and ensured compatibility with the necessary libraries and tools.

#### 4.4.2. Mixture of Experts (MoE) Model Training

As depicted in Figure 6B, the loss curves for the MoE model show similar trends. The training loss decreases consistently, while the validation loss stabilizes after an initial rapid decrease, indicating effective learning and generalization.

### 4.5. Maintenance and Experimental Setup for C. elegans

#### 4.5.1. Culturing Worms

The wild-type *Caenorhabditis elegans* strain N2 was obtained from the *Caenorhabditis* Genetics Center (Minneapolis, MN, USA) and maintained on nematode growth medium (NGM) agar plates. NGM was prepared by dissolving 3 g NaCl, 2.5 g BactoPeptone, and 20 g agar in 1 L of double-distilled water (ddH_2_O). After autoclaving, the medium was cooled to approximately 55–60 °C, and supplemented with 25 mL potassium phosphate buffer (PPB), 1 mL of 1 M MgSO_4_, 1 mL of 1 M CaCl_2_, and cholesterol solution (5 mg/mL). Plates were poured immediately after mixing.

Worm synchronization was performed using the standard bleaching protocol. Briefly, gravid adults were treated with a solution containing ddH_2_O, 5 M NaOH, and 10% sodium hypochlorite (NaClO), which dissolves adult bodies while leaving eggs intact.

#### 4.5.2. Toxicity Assay of Test Compounds in *C. elegans*

The age-synchronous L4-larval nematodes are collected in K-medium quickly. The nematode suspension is allowed to settle for 10 min, and the supernatant containing the *E. coli* is aspirated. The remaining suspension is diluted to approximately ~40 L4-larval nematodes/100 μL with K-medium. 100 μL of chemical solution is added to each well of a 96-well plate and mixed.

Exposures were conducted for 24 h at 20 °C without food supplementation. Following treatment, worms were counted under a microscope. Mortality was scored immediately after exposure by assessing the absence of tactile response to gentle prodding with a platinum wire. Worms failing to respond were recorded as dead.

Live worms and dead worms were manually counted and recorded as N_0_ and N, respectively. Survival rate was calculated using the formula:

$$Survival\;rate=\frac{N_{0}}{N_{0}+N}\times 100\%$$

All chemicals were purchased from Sigma (Shanghai, China) unless otherwise specified.

## 5. Conclusions

In conclusion, the HazChemNet model represents an advancement in the field of chemical informatics, offering a framework for predicting the hazardousness of chemical compounds. By combining attention mechanisms and the MoE model, we have developed a method that not only achieves high predictive performance but also holds substantial potential for practical applications in chemical safety assessment.

## Figures and Tables

**Table 1:** Comparison of the proposed HazChemNet model with traditional machine learning algorithms (SVM, Random Forest, and Logistic Regression) for hazardous chemical classification. The performance metrics include accuracy, precision, recall, F1-score, and AUC.

Model	Accuracy (%)	Precision (%)	Recall (%)	F1-Score (%)	AUC (%)
HazChemNet	91.9 ± 1.3	88.9 ± 2.0	94.0 ± 1.2	91.4 ± 1.3	92.9 ± 1.1
SVM (Support Vector Machine)	88.4 ± 2.0	85.6 ± 2.3	89.7 ± 2.1	87.6 ± 2.0	90.3 ± 1.5
Random Forest	89.2 ± 1.8	86.5 ± 2.2	90.0 ± 2.0	88.2 ± 1.9	91.1 ± 1.4
Logistic Regression	85.6 ± 2.5	82.3 ± 3.0	86.0 ± 2.8	84.1 ± 2.4	87.5 ± 1.8

**Table 2:** Performance Impact of Ablated Components.

Model Variant	Accuracy	Precision	Recall	F1 Score
Full Model	91.9 ± 1.3	88.9 ± 2.0	94.0 ± 1.2	91.5 ± 1.3
Without MolLogP	90.5 ± 1.5	87.8 ± 2.2	92.5 ± 1.4	90.0 ± 1.4
Without MolWt	90.0 ± 1.6	87.5 ± 2.3	92.0 ± 1.5	89.5 ± 1.5
Without NumHDonors	89.8 ± 1.7	87.3 ± 2.4	91.8 ± 1.6	89.4 ± 1.6
Without NumHAcceptors	89.3 ± 1.8	87.0 ± 2.5	91.5 ± 1.7	88.8 ± 1.7
Without Morgan Fingerprint	85.0 ± 2.0	82.0 ± 3.0	88.0 ± 2.0	84.5 ± 2.0

**Table 3:** LD_50_ of compounds.

Compounds	Hazardous	LD_50_ (mg/mL)
Ethyl Alcohol	haz	46.4–100
Atropine	haz	21.5–46.4
Lead Acetate	haz	0.464–1
Glycerol	non-haz	/
Gibberellin	non-haz	/
Citric Acid	non-haz	/

## Data Availability

All relevant data are included in the Supplementary of this article.

## Supplementary Materials

The following supporting information can be downloaded at: https://www.mdpi.com/article/10.3390/ijms26199288/s1.
