# Supplementary material for: HazChemNet: A Deep Learning Model for Hazardous Chemical Prediction
Source: Int J Mol Sci. 2025 Sep 23;26(19):9288. doi: 10.3390/ijms26199288 (PMC12524297; doi:10.3390/ijms26199288)
Supplement: Supplementary file 1 [file ijms-26-09288-s001.zip › Supplementary_Table_and_Figure.pdf]

Table S1. Cross-Validation Performance.

Fold	Accuracy (%)	Precision (%)	Recall (%)	F1-Score (%)	AUC (%)
Fold 1	92.3 ± 1.2	89.5 ± 2.3	94.7 ± 1.4	92.0 ± 1.3	93.2 ± 1.1
Fold 2	91.5 ± 1.3	88.7 ± 2.1	93.2 ± 1.2	91.0 ± 1.5	92.5 ± 1.0
Fold 3	93.0 ± 1.1	90.2 ± 1.7	95.0 ± 1.0	92.5 ± 1.1	94.1 ± 0.9
Fold 4	90.8 ± 1.5	87.9 ± 2.2	92.8 ± 1.3	90.5 ± 1.4	91.7 ± 1.2
Fold 5	92.0 ± 1.4	88.3 ± 2.0	94.0 ± 1.1	91.7 ± 1.2	92.9 ± 1.0
Mean ± SD	91.9 ± 1.3	88.9 ± 2.0	94.0 ± 1.2	91.5 ± 1.3	92.9 ± 1.1

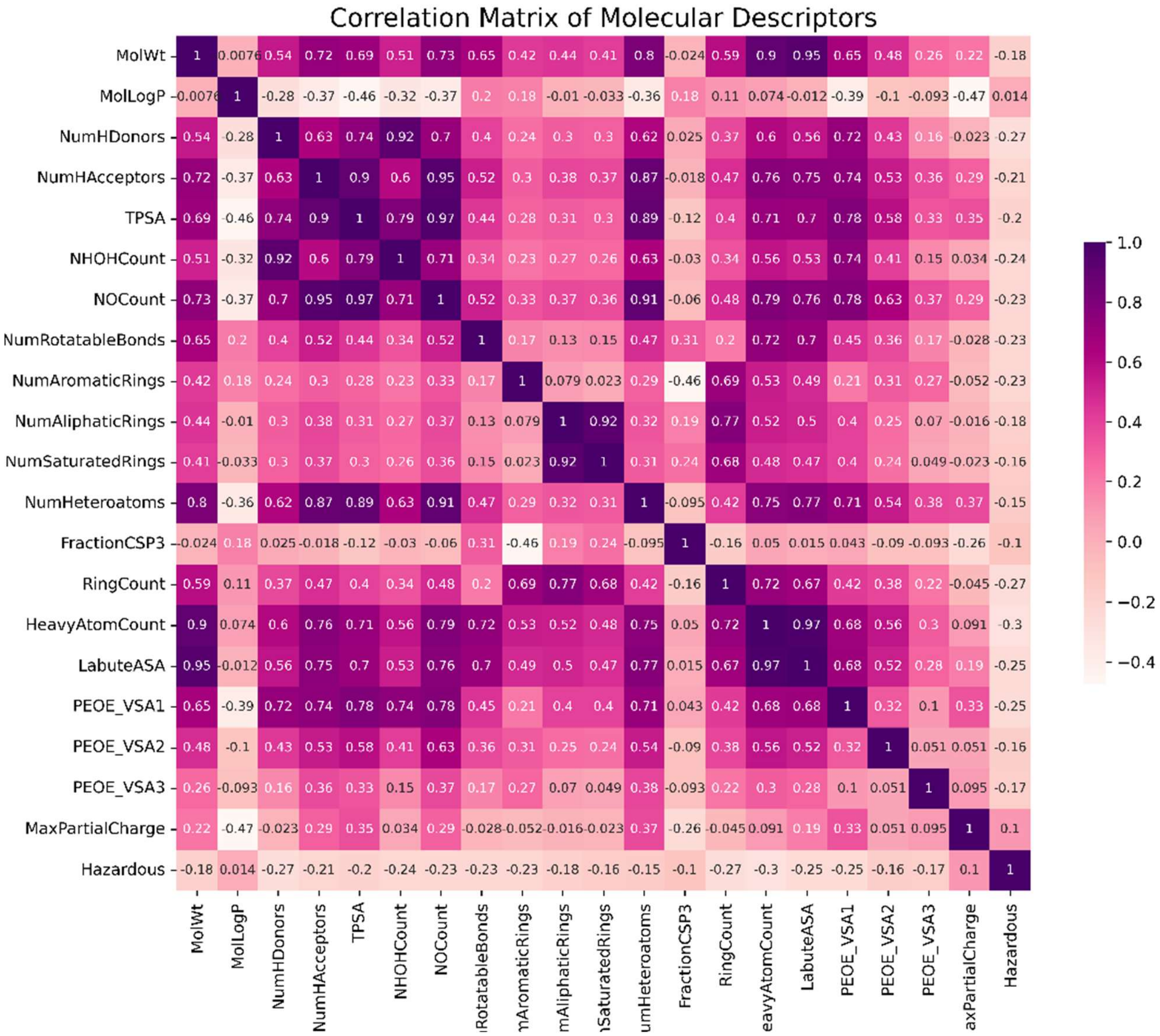

Figure S1. The pairwise correlations between 20 molecular descriptors used in the model for screening hazardous chemicals. The color scale ranges from -1 (perfect negative correlation) to 1 (perfect positive correlation), with 0 indicating no correlation. The matrix helps identify highly correlated features, which can be useful for feature selection and understanding the relationships among the descriptors.
